# Electroanalysis of Fentanyl and Its New Analogs: A Review

**DOI:** 10.3390/bios12010026

**Published:** 2022-01-05

**Authors:** Marta Katarzyna Choińska, Ivana Šestáková, Vojtěch Hrdlička, Jana Skopalová, Jan Langmaier, Vítězslav Maier, Tomáš Navrátil

**Affiliations:** 1 J. Heyrovský Institute of Physical Chemistry of the Czech Academy of Sciences, Dolejškova 3, 182 23 Prague, Czech Republic; marta.choinska@jh-inst.cas.cz (M.K.C.); vojtech.hrdlicka@jh-inst.cas.cz (V.H.); jan.langmaier@jh-inst.cas.cz (J.L.); tomas.navratil@jh-inst.cas.cz (T.N.); 2 UNESCO Laboratory of Environmental Electrochemistry, Department of Analytical Chemistry, Faculty of Science, Charles University in Prague, Albertov 6, 128 43 Prague, Czech Republic; 3 Department of Analytical Chemistry, Faculty of Science, Palacký University in Olomouc, 17. Listopadu 12, 771 46 Olomouc, Czech Republic; vitezslav.maier@upol.cz

**Keywords:** fentanyl, fentanyl analogs, amperometry, voltammetry, screen-printed electrode, metabolite, oxidation

## Abstract

The review describes fentanyl and its analogs as new synthetic opioids and the possibilities of their identification and determination using electrochemical methods (e.g., voltammetry, potentiometry, electrochemiluminescence) and electrochemical methods combined with various separation methods. The review also covers the analysis of new synthetic opioids, their parent compounds, and corresponding metabolites in body fluids, such as urine, blood, serum, and plasma, necessary for a fast and accurate diagnosis of intoxication. Identifying and quantifying these addictive and illicit substances and their metabolites is necessary for clinical, toxicological, and forensic purposes. As a reaction to the growing number of new synthetic opioid intoxications and increasing fatalities observed over the past ten years, we provide thorough background for developing new biosensors, screen-printed electrodes, or other point-of-care devices.

## 1. Introduction

Opioids are an important group of substances with the ability to bind to opioid receptors (in the periphery and the central nervous system μ−, κ−, and δ− receptors) [1]. Besides opium (obtained from *Papaver somniferum*), many known semi-synthetic and synthetic substances can interact with opioid receptors. Opioids have been studied primarily as drugs for their analgesic activity in successful pain relief and have belonged among the safest analgesics. Semi-synthetic and synthetic opioids used for treatment (e.g., codeine, hydrocodone, oxycodone, fentanyl and its analogs, tramadol, tapentadol, etc.) are very effective drugs for managing severe acute pain, cancer pain, and, in some cases, chronic non-tumor pain [2,3,4].

During the last decade(s), the illegal chemical modifications of semi-synthetic and synthetic opioids (namely fentanyl) and synthesis of new non-fentanyl opioid receptor substances have intensified. These substances, abused as illicit drugs, belong to the group of the novel synthetic opioids (NSOs), appear in the list of New Psychoactive Substances (NPSs) issued by the United Nations Office on Drugs and Crime (UNODC) [5,6,7]. The abusing of semi-synthetic opiates (as codeine, oxycodone, and hydrocodone) is currently receding into the background, while the abuse of NSOs is increasing rapidly (namely fentanyl and its analogs, non-fentanyl opioids, such as U-47000, AH-7921, MT-45) [8]. Low production costs and euphoric properties (as with many other opioids, such as morphine, heroin, codeine) often lead to NSOs usage in mixed street drugs, for example, with heroin or other NPSs, such as synthetic cannabinoids [9,10,11]. Fentanyl analogs (fentanyls) are also available as “research chemicals”, “legal highs”, “herbal baths”, and “bath salts” on the Internet.

**Figure 1:**
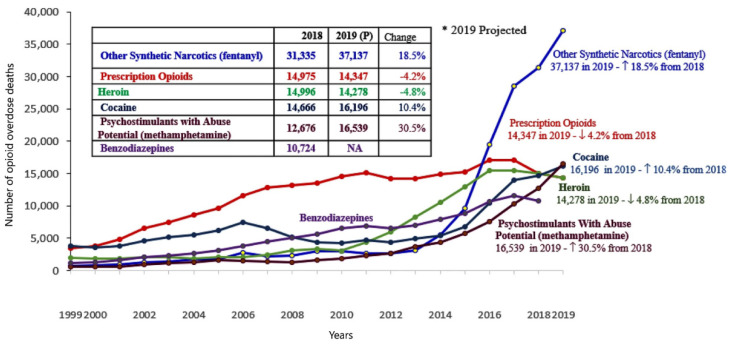
The number of fentanyl, fentanyl analogs, and other opioid overdose deaths in 1999–2019. Reprinted with permission from ref. [16] under Creative Commons Attribution License 4.0, 2021.

The most common forms of abuse include smoking, intranasal application, transdermal administration, sniffing, vaping, oral administration, and injection [12]. Hundreds of deaths from the misuse of fentanyl and its derivatives have been reported since the beginning of these drugs abuse in 1993. According to WHO estimates, approximately 115,000 people died of opioid overdose in 2017 [13]. The fentanyl and fentanyl analogs “epidemic” has been summarized recently [8,12,14,15]. Moreover, during the coronavirus pandemic, the number of prescribed drug intake and overdosing increased, as well as intake of illicit drugs, especially opioids [8,16,17,18]. These increasing trends are shown in Figure 1.

In 2016–2021 (the last information from June 2021), in the Czech Republic, 79 cases of fentanyl intakes were reported to the Czech Toxicological Information Centre [19]. 40% of them were in the toxic dose range and 6% in the lethal dose range. Moreover, almost 30% of cases were intentional drug abuses to commit suicide [19]. This brief statistical overview is in good agreement with data described in the literature, which confirm that opioid intoxications are a global problem [8,12,16,17,20,21,22,23,24].

Notably, non-fatal opioid overdoses are several times more common than fatal ones. The risk is associated with dose-dependent respiratory depression, a common cause of death in the case of fatal intoxication [5,9,10,25,26]. The other symptoms of NSOs intoxication include, e.g., nausea, vomiting, constipation, sedation, cognitive impairment, and pruritus [3].

The increasing number of cases of NSO abuse is putting pressure on the development of analytical methods not only for the accurate identification of individual parent components of abused drugs but also for sufficiently sensitive analytical methods for the determination of originally abused substances and their metabolites. The doses of abused NSOs are relatively low (e.g., for acetylfentanyl 3–5 mg per os), compared to other typically abused illicit drugs (amphetamines, cocaine, etc.). Thus, expected concentrations in biological samples (serum, plasma, urine, vitreous humor, etc.) are in ng mL^−1^ to pg mL^−1^ range (less than 100 nmol L^−1^). Moreover, NSOs also possess a short biological half-life (typically only several hours). In this respect, knowledge of NSOs metabolism and the possibility of sensitive determination of metabolites in biological matrices can extend the detection window. Determining the ratio of metabolite-to-parent drug can also distinguish between acute or delayed death [27].

The review focuses on the determination of fentanyl and its derivatives and other non-fentanyl opioids by electrochemical methods alone and by hyphenation of electrochemical methods with high-performance liquid chromatography (HPLC) and electromembrane extraction in various types of matrices (biological matrices and other liquid or solid matrices).

### 1.1. Fentanyl and Fentanyl Analogs Used for Human and Veterinary Medical Purposes

**Figure 2:**
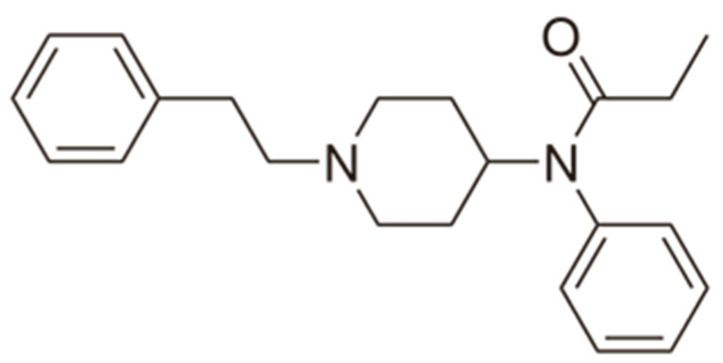
Fentanyl structure (C_22_H_28_N_2_O).

Fentanyl, *N*-phenyl-*N*-[1-(2-phenylethyl)piperidin-4-yl]propenamide, CAS No. 437-38-7 (Figure 2), and its derivatives (the most common are listed in Table 1) have been widely used primarily as medications (synthetic opioids).

Fentanyl was first synthesized by Paul Janssen in 1959 (or 1960) [28,29,30]. It is structurally close to pethidine (meperidine)—another synthetic opioid used as a pain killer. Fentanyl is known as a synthetic narcotic analgesic, which can act as an agonist of opioid receptors [9], being about 100 times more potent than heroin [9] or morphine [31]. Fentanyl and its analogs with piperidine-based structures have significantly different structures than morphine and other semi-synthetic opioids (e.g., codeine, hydrocodone, oxycodone, buprenorphine, methadone, etc.). Moreover, it has fewer adverse effects than morphine or pethidine [10]. Thanks to these advantages and relatively small dosages needed, they are often used for anesthesia (namely in surgical settings), treatment of (chronic) pains, and supplemental medications for breakthrough pain in cancer patients. In the average person, anesthesia is achieved after 25–125 mg of fentanyl [12]. Moreover, fentanyls stabilize cardiovascular activity (even in patients under critical conditions [10]), increasing their medical use. Fentanyl belongs to the most potent opioids available for human medical use [5].

Fentanyl is commercially available as a water-soluble drug in hydrochloride or citrate form [10]. According to various literature sources, fentanyl dissociation constant p*K*_A_ amounts to between 8.4 and 9.0 (Table 2), thus making it partially unionized in blood and bound to specific compounds, such as erythrocytes, albumins, and other endogenous compounds. These values are relatively close to those of morphine (p*K*_A_ = 8.08) or fentanyl analog sufentanil (p*K*_A_ = 8.51) [32]. Fentanyl is highly lipophilic (log*P* 2.3 octanol/buffer pH 7.4 [33]) and can enter the central nervous system (CNS) 100 times more easily than morphine [10]. Unfortunately, it has also been used since the 70’s as an illicit street drug individually or in a mixture with other illicit drugs, such as heroin or synthetic cannabinoids.

Fentanyl analogs were synthesized to develop new opioid drugs with better pharmacological properties and fewer side effects. Despite a large number of available fentanyl analogs, only three have been approved for human medical use, i.e., sufentanil, alfentanil, and remifentanil [5,34]. The administration is limited to intravenous anesthesia or severe pain treatment. Compared to fentanyl, its analogs may have slightly different metabolic pathways, physiological activity, and properties. For example, alfentanil has a smaller volume of distribution than fentanyl, less solubility in lipids, and a shorter elimination half-life [29]. As a result, it has lower potency than fentanyl and has been widely used in medicine [35]. Sufentanil was reported as 5–10 times more potent than fentanyl, and its interactions are more rapid [35]. Another one, carfentanyl, has been approved for veterinary use in the case of large animals [5,35]. Carfentanyl is 10,000 times more potent than morphine as an analgesic.

Physicochemical parameters of fentanyl and its chosen derivatives (used in human and veterinary medicine or misused as illicit drugs) are summarized in Table 2.

Many other fentanyl derivatives have been described since the 1960s, but they have not been used in human or veterinary medicine. The new non-therapeutic fentanyl analogs have been later described as NPSs or NSOs, seriously affecting the neurological system [34]. New fentanyl analogs are reported from year to year; for example, three of them appeared on the European market in 2018: 3-methylcrotonylfentanyl, furanylbenzylfentanyl, and 4-fluorocyclopropylbenzylfentanyl (structure shown in Table 1) [40].

There are many non-therapeutic fentanyl analogs reported to the United Nations Office on Drugs and Crime Early Warning Advisory (UNODC EWA), which are common in Europe, Asia, and America [5,29,40]. The United States Drug Enforcement Agency (DEA) listed fentanyl and its derivatives (isomers, thioethers, and salts) in the Schedule I list [41]. Table 1 summarizes the most popular fentanyl analogs in 2012–2018 used either for medical treatment or appeared as illicit street drugs. Metabolism and toxicological aspects of fentanyl and other NSOs derived from fentanyl are discussed below.

### 1.2. Metabolism of Fentanyl and Fentanyl Analogs

The knowledge of the metabolism of fentanyl and its analogs is essential for the identification of administered substance(s) in various body fluids ante- and post-mortem (namely blood, plasma, serum, urine, cerebrospinal fluid, vitreous humor, and organs) after acute or chronic intoxication or death. The study of these processes is critical due to their short biological half-life (units of hours in case of oral or intravenous applications) and consequently the short detection window of the parent fentanyl(s) in the blood. Identification of characteristic metabolites, first of all in urine, may contribute to the exact identification of parent substance(s).

While metabolites of morphine are pharmacologically active, metabolites of fentanyl and medically-approved fentanyl derivatives are mostly inactive [4]. Fentanyl has several sites, which can take part in its metabolic transformation. Fentanyl is metabolized mainly in the human liver to the main metabolite norfentanyl (26–55% of fentanyl dose is excreted as norfentanyl). This principal metabolic pathway is caused by cytochrome CYP3A4 [4], together with CYP3A5 and CYP3A7 isoenzymes, through the oxidative *N*-dealkylation of the fentanyl’s piperidine ring [42]. The same *N*-dealkylation takes place in duodenal microsomes [26,29]. Other possible metabolic reactions transform fentanyl into hydroxyfentanyl, hydroxynorfentanyl, and despropionylfentanyl. These metabolites can take part in the subsequent biotransformation at enzyme catechol-*O*-methyltransferase to the secondary metabolic products [29]. Other metabolic pathways change fentanyl into hydroxypropionylfentanyl, hydroxypropionylnorfentanyl, or despropionylfentanyl. Metabolites are mainly present in saliva, urine, stool, and human plasma [29]. About 70% of the administered dose is excreted in the urine in 72 h (mostly in the form of metabolites), and about 10 to 20% of the administered dose is excreted unchanged in 48 h. Only 8–10% of unchanged fentanyl is released from the body through the renal or fecal pathway [26]. Fentanyl crosses the placenta, and small amounts may be found in breast milk, too.

As mentioned above, three fentanyl analogs (alfentanil, sufentanil, and remifentanil) are widely used in anesthesia and pain treatment. Alfentanil and sufentanil are metabolized similarly to fentanyl via the hepatic pathway to identical *N*-dealkylated products (norsufentanil, noralfentanil). From this point of view, the administered alfentanil or sufentanil cannot be distinguished using the methods which detect products of metabolic transformation only [26,29]. Norsufentanil exhibits some bioactivity, the other product of sufentanil metabolism demonstrates about 10% of the original sufentanil activity only, which is too small to be clinically significant [29].

Remifentanil is mostly metabolized directly in the blood by non-specific esterases located in erythrocytes (95%) and has a very short time of activity [43,44]. Its main metabolite, remifentanil acid, is practically non-active and is removed from the body via the renal pathway with an elimination half-life of approximately 90 min [26,29,43,44]. It is the only analog that is metabolized by non-CYP enzymes [26].

Carfentanyl is one of the well-known fentanyl analogs used in veterinary medicine, which shows potency 30–100 times higher than fentanyl itself. According to our literary research, there has not been a published study about metabolic pathways of this drug in vivo in humans. Identification of twelve metabolites of carfentanyl was done using human liver microsomes and human hepatocytes [22,29,45]. *N*-demethylation as the main biotransformation was predicted in silico and confirmed by high-resolution mass spectrometry. Carfentanyl and its metabolite, norfentanyl, may accumulate in the human body, which can cause resistance to the antidote [22].

**Figure 3:**
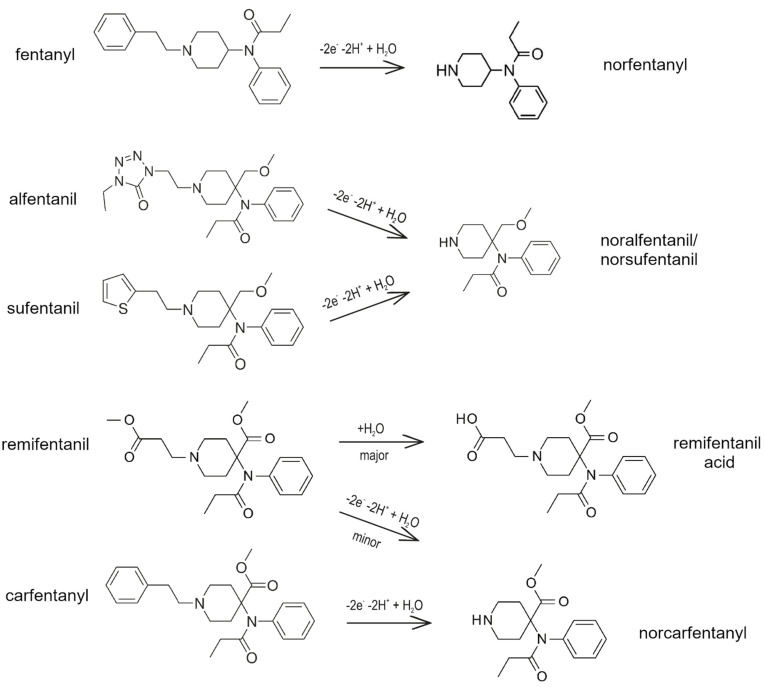
Proposed main metabolic mechanisms for fentanyl and its four analogs used in human and veterinary medicine, on the base of ref. [22,29,44,45,49].

Figure 3 presents the proposed mechanism of metabolism of fentanyl and its four medicinally-used analogs (alfentanil, sufentanil, and remifentanil) and veterinary medicine (carfentanyl).

Furanylfentanyl, an illegal drug, is another analog with seven times higher potency than fentanyl. Its four metabolites have been found in urine samples taken from intoxicated patients. In almost all cases, hydrolysis product 4-anilino-*N*-phenethylpiperidine (4-ANPP) and its sulfate conjugate were found. A unique metabolite, formed by dihydrodiol formation of the heterocyclic furanyl moiety, was observed in 86% of cases [29,46]. On the other hand, *N*-dealkylated metabolite norfuranylfentanyl was detected only rarely [47].

The pharmacological properties of acetylfentanyl (street names: “China town” or “Synthetic heroin”) are similar to heroin. Up to date, 32 metabolites of acetylfentanyl have been identified in vivo. The main metabolite is *N*-dealkylated product noracetylfentanyl. The next metabolizations of noracetylfentanyl include hydroxylation followed by glucuronidation or sulfation. Dihydroxylation was also confirmed, followed by glucuronidation or sulfation. The other metabolite reactions include monohydroxylation and carbonylation, dihydrodiol formation, dihydroxylation with methylation at the phenyl ring followed by glucuronidation or sulfation, and amide hydrolysis, followed by hydroxylation [47].

Ocfetanyl is a 200 times more potent derivative than morphine. Its biotransformation starts with *O*-demethylation forming the main metabolite. The following reactions include hydroxylation and glucuronidation of *O*-demethylated ocfentanyl.

Butyrfentanyl is 7 times more potent than morphine. The work of Staehali et al. describes two main metabolites hydroxy-butyrfentanyl and carboxybutyrfentanyl in vivo after fatal intoxication by butyrfentanyl [48].

Acrylfentanyl is abused alone or mixed with other drugs. Nor-acrylfentanyl, formed by *N*-dealkylation at the piperidine nitrogen, has been identified as the main metabolite of acrylfentanyl. Other metabolites have been identified, such as monohydroxy- and dihydroxy-derivatives. Monohydroxylation and dihydroxylation occurred at both the phenyl ring and the phenethyl moiety. Three glucuronides have been identified in urine samples without hydrolysis [47].

Cyclopropylfentanyl undergoes extensive metabolism. Eleven metabolites of cyclopropylfentanyl have been identified in the pooled hydrolyzed and non-hydrolyzed human urine of drug abusers. Cyclopropylfentanyl and norcyclopropylfentanyl have been detected in non-hydrolyzed urine. Other metabolites, together with norcyclopropylfentanyl, were conjugated and observed after urine hydrolysis. Cyclopropylfentanyl was detected in both samples, and hydrolyzed, as well as non-hydrolyzed, respectively. Major metabolites in the hydrolyzed samples were 4-hydroxyphenethyl cyclopropylfentanyl (mostly conjugated), 4-hydroxy-3-methoxyphenethyl cyclopropylfentanyl (mostly conjugated), phenethyl dihydrodiol cyclopropylfentanyl, and norcyclopropylfentanyl. Norcyclopropylfentanyl was the most abundant metabolite in the non-hydrolyzed samples [50].

The details about the metabolism of other fentanyl derivatives have been discussed in the review by Wilde et al. [29] and the research article by Watanabe et al. [47].

### 1.3. Toxicology of New Synthetic Opioids

As mentioned above, fentanyl and its analogs react similarly to morphine with the receptors in the CNS. Their exact toxicokinetics and toxicodynamics have not been deeply understood for a long time. However, this knowledge could be critical for clinical procedures. Taking fentanyl or fentanyl analogs repeatedly, whether as prescribed or for nonmedical reasons, increases the risk of addiction, dependence, and tolerance. Concerning that fact, the new study of two fentanyl derivatives toxicology was published [51].

4-Fluorocyclopropylbenzylfentanyl (cyclopropanoyl-1-benzyl-4′-fluoro-4-anilinopiperidine, 4F-Cy-BAP, Table 1) and furanylbenzylfentanyl (furanoyl-1-benzyl-4-anilinopiperidine, Fu-BAP, Table 1) can affect morphine receptors to a limited extent. It was also found that 4F-Cy-BAP was much more stable than Fu-BAP. Their metabolism pathways are complicated, and 7 and 15 products have been found for 4F-Cy-BAP and Fu-BAP, respectively. As with fentanyl, the metabolic transformation was mainly generated by the CYP3A4 isozyme. In conclusion, the drug-to-drug interactions and simultaneous intake of other substances can affect isozyme and cause accumulation of fentanyls [4,51].

In addition, antinociceptive effects and neurotoxicity of fentanyls depend on the substitution at the 3- or 4- position of the piperidine ring. It was revealed that the exact effect has a similar mechanism in both cases: medical and intoxicating. This research also confirmed that substitution at positions 3 and 4 strongly affect the activity of fentanyl analogs [35].

## 2. Determination of Fentanyl and Analogs

As stated above, fentanyl and its analogs are commonly used as illicit drugs, especially in mixtures with heroin. Unfortunately, there are some challenges in their proper identification and determination [18]. These difficulties are caused by complicated mixtures with many interferents (frequently of similar chemical structures [52]) and a lack of available analytical standards. Moreover, fast changes in available psychoactive substances on the illicit market could be problematic [53]. To face these challenges, it is important to develop new methods of detection and determination of synthetic opioids.

Due to the high risk of fentanyl and its analogs usage in medical treatments and their abuse as illicit street drugs, there is an urgent need to develop proper determination and validation procedures, both for toxicological and medical purposes. Many different analytical methods have been introduced during the last few years to determine fentanyl and its analogs, e.g., enzyme-linked immunosorbent assay (ELISA) [54], high-performance liquid chromatography (HPLC) [53,55,56,57], or gas chromatography (GC) [53,58]. Other techniques, such as colorimetric detection, Raman, IR, or NMR spectroscopy, are described in the literature, as well [24,59,60].

Especially, chromatographic techniques coupled with mass spectrometry (MS) have been broadly used for fentanyls determination in real samples. This method is often chosen for complex real samples. Proper determination of all sample components often requires separation, selective, and sensitive detection. HPLC-MS methods meet mostly these requirements [53,55,56,57,59]. The other techniques used for the extraction of fentanyls from biological samples are solid-phase extraction (SPE) and liquid-liquid extraction (LLE). LC-MS/MS has been used as a fast and efficient technique with limits of detection (*LODs*) of 0.01 µg·L^−1^ for many fentanyl analogs [61].

Immunoassays have been used for selective determination of fentanyl and butyrfentanyl, as well, especially as a first-step analysis. It can be used even by non-qualified staff, which is important in medical and toxicological procedures [61]. The affinity of various fentanyl metabolites to the fentanyl antibody varies significantly [62]. Immunoassay test ELISA was used, e.g., for batch analysis of real samples in Forensic Toxicology Laboratory (FTL) at the Rhode Island State Health Laboratories. The samples were collected from dead humans after overdosing on illicit drugs containing fentanyl analogs. This test is very sensitive and can be performed using ELISA kits, which are readily commercially available. The drawbacks of this method are lower specificity and possible false-positive results; therefore, another method is often necessary to confirm the result [54].

For the analysis of extremely complicated biological matrices, including blood or cerebrospinal fluid, the elaborate multi-step separation methods are often irreplaceable. Unfortunately, many of the aforementioned methods are expensive, time-consuming, or require complicated instrumentation or preparation procedure [63,64]. Many other matrices, such as pharmaceutical samples, drug preparation, tap water, saliva, or even blood plasma, can be successfully analyzed using simpler methodology and instrumentation. Therefore, the aim to develop a fast, inexpensive, and simple procedure, with high efficiency and low *LOD*, is quite clear. The electroanalytical methods exhibit such advantages, which are attractive for medical and pharmaceutical analyses of synthetic opioids [11,59,63,64]. The possibility to modify the electrode surfaces can further improve the selectivity and sensitivity. Furthermore, electrochemical detectors can be coupled with flow systems, HPLC, or electrophoresis [59].

## 3. Electroanalytical Methods

The overview of electroanalytical methods used for determinations of fentanyl and its derivatives is summarized in Table 3. The most relevant are described in detail in the following paragraphs. The vast majority of the presented methods are especially suitable for trace fentanyl determination in simpler matrices. Commonly available glassy carbon or mercury electrodes can be readily employed in many laboratories worldwide. When cross-contamination is an expected issue, methods using disposable screen-printed electrodes are available, too. Furthermore, sophisticated chemically modified electrodes can solve extreme sensitivity requirements or signal separation between similar fentanyl analogs. A wide range of matrices and concentration ranges are covered by various electrochemical methods, including potentiometry, direct or stripping voltammetry, or HPLC with electrochemical detection.

### 3.1. Adsorptive Stripping Voltammetry

Fentanyl and its derivatives can be adsorbed at the surface of a static or hanging mercury electrode [65,66]. Under optimized conditions (0.05M NaOH, 10% ethanol, preconcentration time 10 min), the *LOD*s of 50 nmol L^−1^ for fentanyl [66] and of 5 nmol L^−1^ for N,N′-bis(1-phenylmethyl-4-piperidinyl)-ethane-diamide (DBPPE), i.e., one of fentanyl derivatives [65], were reached. It was also confirmed that two electrons are transferred during the electrochemical reduction, corresponding to the reduction of the carbonyl group [66]. However, the adsorption of fentanyl and its derivatives to electrode surfaces implies possible fouling effects, especially at metallic-based or metallic film or nanoparticle-modified electrodes.

### 3.2. Differential Pulse Voltammetry

**Figure 4:**
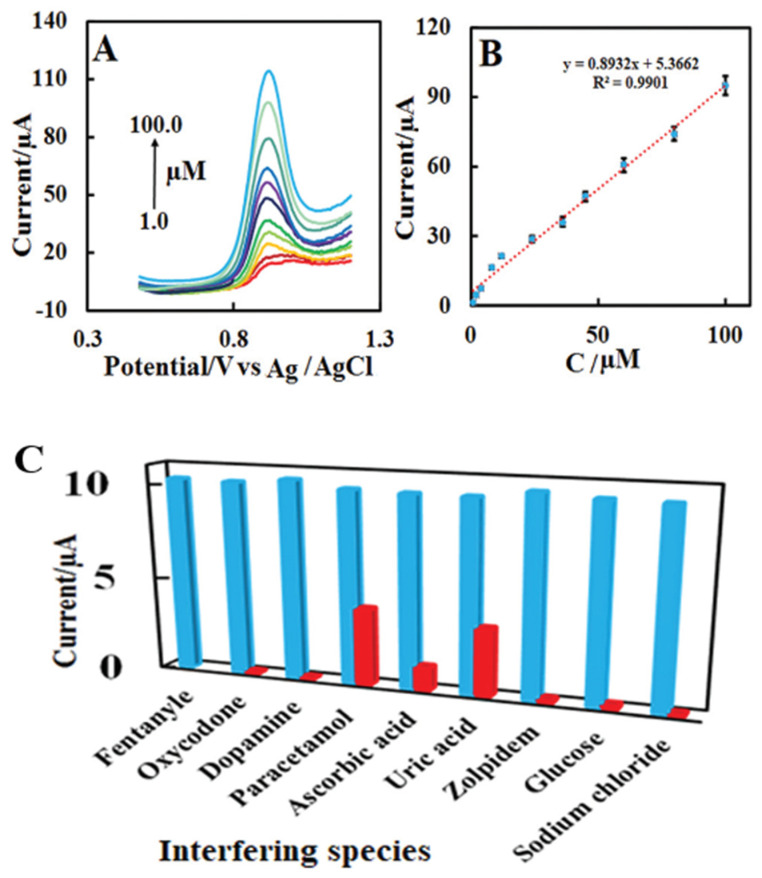
(**A**) Differential pulse voltammograms recorded at screen-printed carbon electrodes modified with zinc-based metal-organic frameworks in presence in 0.1 mol L^−1^ phosphate buffer solution (pH = 7) containing 1–100 µmol L^−1^ of fentanyl with scan rate 50 mV s^−1^. Electrode surface areas (*A*, cm^2^) for the bare SPCE and S Zn(ii)-MOF/SPCE were estimated to be 0.12 cm^2^ and 0.48 cm^2^, respectively. (**B**) Linear dependence of peak currents (*I*_p_) on fentanyl concentrations. (**C**) Detection of 10 µmol L^−1^ of fentanyl and common interfering species in 0.1 mol L^−1^ phosphate buffer (pH = 7). Reprinted with permission from ref. [67]. Copyright 2021 Royal Society of Chemistry.

Recent research on the determination of fentanyls in urine and plasma samples was aimed at the use of screen-printed carbon electrodes (SPCEs) modified with zinc-based metal-organic frameworks (MOFs). Zn(II)-MOF proved to be an effective and stable modifier with a large surface and extremely high porosity. Differential pulse voltammetry (DPV) was used to determine fentanyl concentrations in a wide range (linear dynamic range (*LDR*) 1–100 µmol L^−1^) and offered good selectivity and repeatability (Figure 4). The small effect of interferents and relatively low *LOD* (0.3 µmol L^−1^) have shown that the developed method is a promising tool for fentanyl determination. Low cost, fast, and simple production are the other added benefits of this electrode type [67]. Moreover, the screen-printed design allows its use as disposable sensors to avoid cross-contamination.

Differential pulse adsorptive stripping voltammetry at the glassy carbon electrode (GCE) modified with multi-walled carbon nanotubes (MWCNTs) was also used for fentanyl determination in human urine and blood serum. Cyclic voltammetry (CV) at bare GCE and MWCNTs-GCE was used for the investigation of the electrochemical behavior of fentanyl in phosphate buffer solution. The modified electrode exhibits significant catalytic activity towards the fentanyl oxidation and provides a better-developed oxidation peak compared to that registered at a bare GCE [68]. Determination of fentanyl at the modified GCE was performed using adsorptive stripping DPV with accumulation time of 500 s. The reported linear range was over three orders of magnitude with *LOD* = 0.1 µmol L^−1^. The method was validated using spiked human urine and human blood serum samples. Recoveries for urine and blood serum were 101% and 103%, respectively, demonstrating clinical usability [68].

#### 3.2.1. DPV at Glassy Carbon Electrode Modified with Carbon Nano-Onions

GCE modified with carbon nano-onions (multi-layer fullerenes) is another recently developed tool applicable for fentanyl determination. Carbon nano-onions are one of the newest nanomaterials and carbon allotropes. They are effective due to the porous layer and spherical aggregates, enabling the formation of a large effective surface area of the electrode. Reported *LOD* is 0.3 µmol L^−1^ [49]. It was also observed that this modification shifts fentanyl peak potential to a more negative value without any influence of common interferents. Wide *LDR* and low cost of preparation are the other advantages [49].

#### 3.2.2. DPV at Single-Walled Carbon Nanotubes Electrode

Single-walled carbon nanotubes (SWCNTs) represent another promising material for electrochemical detection of fentanyl analogs. They have been used to create new electrodes with large surface areas and high conductivity and increased signal-to-noise ratio. CV confirmed the irreversibility of the oxidation/reduction processes of fentanyl analogs. DPV with SWCNTs modified electrodes was found to be more sensitive than CV providing for fentanyls the *LOD*s about 11 nmol L^−1^ [69].

### 3.3. Differential Normal Pulse Voltammetry

Differential normal pulse voltammetry (DNPV) was applied for in vivo investigation of catecholamine metabolism in the rat *locus coeruleus*. Fentanyl or other morphine-like drugs can induce changes in catechol oxidation, which can be monitored electrochemically. The measurements were performed using a carbon fiber electrode. The study using DNPV confirmed that fentanyl causes a significant decrease in catechol oxidation current in rats *locus coeruleus* [78]. On the contrary, according to Reference [79], if DPV is applied, fentanyl increases the catechol oxidation current in rat striatum. Generally, both methods can be used for in vivo monitoring of catecholamine metabolism and brain dynamic activity, as well as for the investigation of mechanisms of opioid influence on brain assessment [78,79].

### 3.4. Square-Wave Voltammetry

Microinvasive instrumentation for simultaneous determination of fentanyl and organophosphate nerve agents was described in Reference [70]. It was designed as a wearable array of microneedle sensors with one of the microneedles serving as a working electrode filled with carbon paste. Square wave voltammetry (SWV) was applied, with relatively high selectivity for the determination of fentanyl and its derivative norfentanyl. No interferences among fentanyl and commonly present substances were registered. At MWCNTs modified electrode, the *LOD* dropped to 50 nmol L^−1^ [70].

#### 3.4.1. Square-Wave Voltammetry with Microcatheter-Based Dual-Analyte Sensor

**Figure 5:**
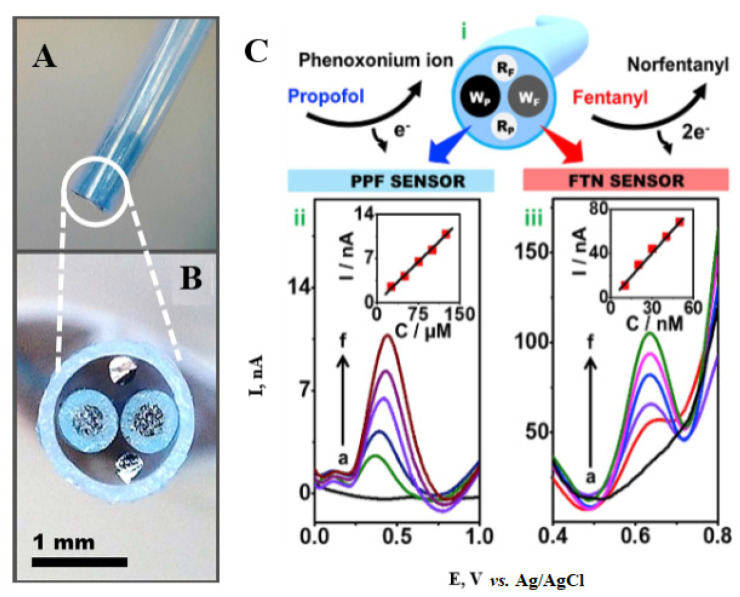
(**A**) Photo of the integrated dual microcatheter sensor. (**B**) Cross-section of the integrated dual microcatheter sensor. (**C**) Concentration dependencies: simultaneous determination of propofol (PPF) and fentanyl (FTN) using the microcatheter sensor. (i) Scheme of simultaneous analysis of PPF and FTN. (ii) SWVs of PPF sensor upon addition of a mixed solution of PPF/FTN (2.5 mmol L^−1^ PPF/1 μmol L^−1^ FTN) in the range of 25–125 μmol L^−1^ in 25 μmol L^−1^ increments (a to f). (iii) SWVs of FTN sensor recorded in artificial plasma solution while adding mixed concentrations of PPF and FTN in the range of 10–50 nmol L^−1^. SWV potential ranges; 0–1000 mV for PPF and 400–800 mV for FTN. Reprinted with permission from ref. [71], Copyright 2021 Elsevier Science & Technology Journals.

SWV with a microcatheter-based dual-analyte sensor was capable of simultaneous real-time continuous monitoring of fentanyl and propofol drugs. It was enabled by quite a low cross-reactivity between these two analytes. The high selectivity of this detector was tested by the addition of common interfering compounds [71]. The proposed method proved to be sufficiently sensitive for analytical purposes with low *LOD*s for fentanyl and propofol (2.18 nmol L^−1^ and 4.3 µmol L^−1^, respectively; Figure 5). Moreover, the sensor is fast to use, selective, exhibits wide *LDRs* in the cases of both analytes, and is sufficiently reliable. Good performance in the analysis of artificial plasma and untreated blood samples allows its use for real-time monitoring of anesthetics during surgical operations [71]. This arrangement demonstrates a huge potential of the application of simple electroanalytical methods in complex matrices for point-of-care use.

#### 3.4.2. Screen-Printed Electrodes Modified with the Room Temperature Ionic Liquid

Two other experimental protocols with screen-printed electrodes have been lately introduced. One of them is based on SPCE modified with the room temperature ionic liquid (RTIL, composed of 1-butyl-1-methylpyrrolidinium bis(trifluoromethylsulfonyl)imide [C_4_C_1_pyrr][NTf_2_]). In this application, cyclic SWV was used to investigate the mentioned drugs. The determination is based on irreversible fentanyl oxidation in the first anodic scan. The product of this oxidation is further analyzed during the following anodic and cathodic scans with reversible characteristics. Using this method, fentanyl could be selectively determined even in a mixture of common interferents, making it useful for fast, sensitive, and selective identification of fentanyl in complicated drug mixtures [15,80].

#### 3.4.3. Screen-Printed Electrodes Printed on Gloves

The other method is based on electrodes printed on gloves. The idea of small, portable measuring equipment based on a wearable glove with flexible electrochemical sensors seems to be very useful for rapid point-of-care identification of fentanyl in street drugs. This method uses two electrodes, one printed on the thumb (sampling) and the other on the index finger (sensing electrode). The electrochemical “cell” is thus created by joining fingers and the signal is transferred to a portable potentiostat on the hand [72]. Using SWV at this wearable sensor, *LODs* of 10 μmol L^−1^ were reached for fentanyl in the liquid and powder forms. The high selectivity of the sensor was also confirmed by the determination of fentanyl in the mixture with possible common interferents. Due to a clear fentanyl response even in the mixture, the technique is applicable in the analysis of street drugs with complicated compositions [15,72].

#### 3.4.4. Unmodified Screen-Printed Electrodes

The recently published method has employed square-wave adsorptive stripping voltammetry (SWASV) at unmodified SPCE. This research has investigated the electrochemical behavior of fentanyl and the mechanism of fentanyl irreversible oxidative conversion to norfentanyl. Under optimum experimental conditions (i.e., buffer pH of 8.5), two oxidation peaks were observed, and two *LDR*s on concentration dependences were found. The first *LDR* was observed at fentanyl concentration below 1 mg L^−1^ leading conclusion that the method might be used for the trace analysis of this drug. The method was also used for a single drop analysis. *LOD*s were (0.037 ± 0.017) mg L^−1^ for the cell method and (0.233 ± 0.025) mg L^−1^ for the single drop analysis, respectively [73]. Some of the commonly present compounds (e.g., caffeine) have interfered with the second fentanyl oxidation peak, while the first one could be successfully used for the drug determination, even in the presence of a mixture of interfering compounds.

In the next research, stripping voltammetry at screen-printed electrodes was used for the determination of fentanyl in the lacrimal fluid. The study shows pharmacological kinetics of this opioid analgesic correlated with the shape and height of the metal signals at the electrode. The background electrolyte showed signals of chosen metal ions (Cd, Pb, Co, Zn). After the addition of the fentanyl solutions with different concentrations, metal ion behavior on the electrode was changed. In the last step, the same procedure was used for the determination of fentanyl in the lacrimal fluids of patients [81].

### 3.5. Cyclic Voltammetry at a Polarized Ionic Liquid Membrane

**Figure 6:**
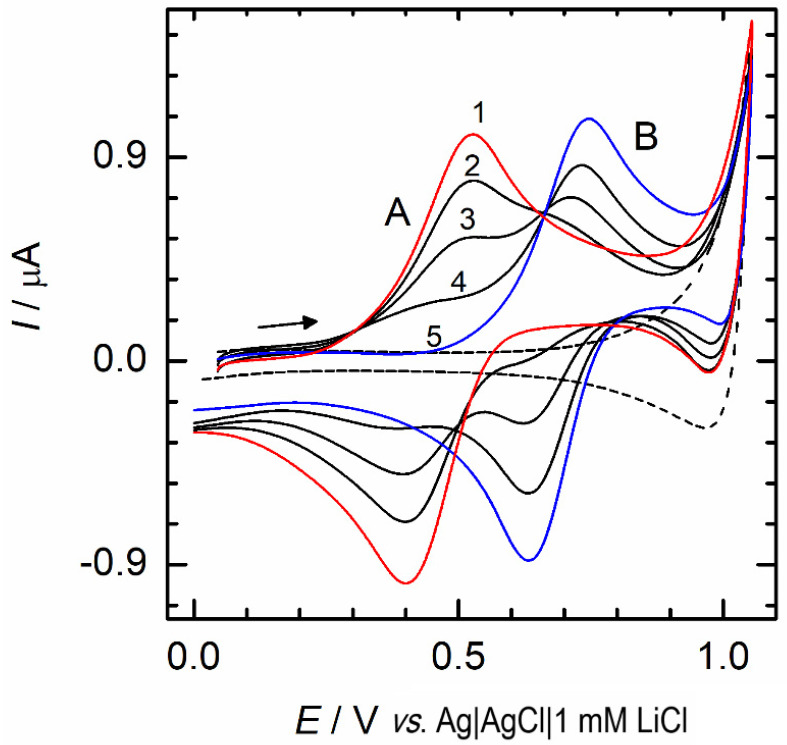
Cyclic voltammograms (10 mV s^−1^) measured using the IL membrane with 1 mmol L^−1^ LiCl (pH 7) in the absence (dashed line) and the presence (solid lines) of fentanyl (peak A) and norfentanyl (peak B) in the aqueous phase at the concentrations 200 and 0 (1), 150 and 50 (2), 100 and 100 (3), 50 and 150 (4), or 0 and 200 (5) μmol L^−1^, respectively. Reprinted with permission from ref. [82]. Copyright 2021 John Wiley and Sons.

One of the newest methods for the determination of fentanyl and its analogs is cyclic voltammetry at the ionic liquid membrane (IL membrane). The ion lipophilicity plays a critical role in the transporting processes responsible for drug accumulation in the human body and is comparable with pharmacological efficiency. Results achieved using cyclic voltammetry (Figure 6) at IL membrane are consistent with the biological behavior of the tested opioids, providing insight into interactions between drugs and biological membranes. In addition, this method enables the calculation of the ion partition coefficients, ion transfer standard Gibbs energy, and the partition coefficients of the neutral opioids. The other advantages of this method consist in the chemical non-destructivity of the process, low consumption of ionic liquid phase, and *LOD* below 1 µmol L^−1^ [82].

### 3.6. Potentiometry

Potentiometry with ion-selective membrane electrodes (ISMEs) is a relatively recently developed method used for fentanyl determination. The membrane was made of polyvinyl chloride with the addition of plasticizer dibutyl phthalate [74]. This method enables selective fentanyl determination without a preceding separation step using a simple device. After preconcentration and optimization of experimental conditions, the *LODs* 5.43 µmol L^−1^ [63] and 6.29 µmol L^−1^ [74] were reached. The method provided sufficient responses and repeatability of the recorded signals during at least three months of the electrode used. Moreover, the whole procedure was fast and simple with sufficient accuracy for pharmaceutical [63] and medical [74] analysis. This ion-selective electrode enables to distinguish only one target ion from others, with no significant interferences observed.

### 3.7. Electrochemiluminescence

Electrochemiluminescence with different glassy carbon-based electrodes and ionic liquid composite paste electrodes (ILCPEs) have been used for the determination of fentanyls, too. Under optimum conditions, the reached *LOD* of fentanyl amounted to 8.5 nmol L^−1^. The system exhibited good repeatability, long-term stability, and wide *LDR* [75,76].

### 3.8. Indirect Amperometry

Another method applicable for the characterization of neurological effects of heroin and fentanyl is based on high-speed amperometry, monitoring brain oxygen levels after heroin and fentanyl intake. It was performed using a commercially available electrochemical oxygen sensor. The recorded *LDR* amounted to 0–50 μmol L^−1^, and the sensitivity varied from 1.1 to 1.9 nA L μmol^−1^. The most common interferents were tested with no significant effect on the signal. It can be concluded that both drugs caused a decrease in oxygen levels in the brain. Regardless of which drug was used, either pure heroin, pure fentanyl, or a mixture thereof, the effect was rapid, but the duration of the effect was different [83,84,85].

## 4. Hyphenated Techniques

Determinations of fentanyls in biological samples have been mostly performed by chromatographic methods with different detectors, e.g., by liquid or gas chromatography with UV/VIS [53,55] or mass spectrometry (MS) [53,56,57,58,86]. Nevertheless, hyphenation of various techniques with voltammetric methods and various electrodes have also been used for these purposes.

### 4.1. Amperometry in Hyphenation with HPLC

HPLC combined with amperometric detection (AD) or diode array detectors (DAD) represents another viable method for the determination of fentanyl and its analogs. Screen-printed graphite macroelectrodes have been utilized for amperometric detection due to their simple design, reproducibility, and low-cost production [9]. The initial part of the study was aimed at electrochemical reactions of the drugs. Fentanyl oxidation was an irreversible process, agreeing to the aforementioned studies [9]. By amperometric detection, real samples could not be analyzed without prior separation due to overlapping signals of different fentanyls and heroin at optimum pH for CV. Therefore, HPLC had to be used to separate the present analytes. Under optimized HPLC conditions, the analytes were properly separated, and finally, a new procedure for their determination was proposed. The HPLC-DAD system was found to be more sensitive than HPLC-AD, with *LOD* values in the range 1.3–8.7 µmol L^−1^ and 0.48–1.48 µmol L^−1^ for HPLC-AD and HPLC-DAD, respectively. Both methods were highly selective, and the tested common substances found in street drugs (as drugs and their admixtures) have not interfered with fentanyl signals [9].

### 4.2. Electromembrane Extraction Combined with Differential Pulse Voltammetry at Modified Carbon Screen-Printed Electrode

**Figure 7:**
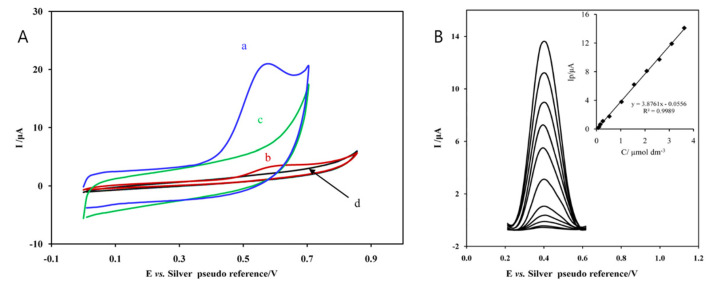
(**A**) CV of 1.3 × 10^−^^4^ mol L^−^^1^ sufentanil in 0.05 M NaCl on (a) MWCNTs-SPCE; (b) bare SPCE; (c) CV of blank at MWCNTs-SPCE; and (d) CV of blank at bare SPCE. (**B**) DP voltammograms of increasing concentrations of sufentanil (down to up: 0.064, 0.10, 0.15, 0.26, 0.52, 1.03, 1.55, 2.07, 2.60, 3.10, and 3.62 µmol L^−^^1^) recorded under optimum conditions. Inset: Concentration dependence of sufentanil in water. Reprinted with permission from ref. [77]. Copyright 2021 Elsevier Science & Technology Journals.

DPV at modified SPCEs with electromembrane extraction (EME) was used for the determination of sufentanil. Electromembrane extraction led to the preconcentration of sufentanil from the sample in the first step by applying a potential across a supported liquid membrane (SLM). Charged drug molecules were extracted from an acidic aqueous sample, through the SLM, into an acidic aqueous acceptor solution (20 μL) which was placed inside the lumen of a hollow fiber. Then, the acceptor solution was analyzed using DPV with cheap commercially available MWCNTs modified SPEs (Figure 7B). Cyclic voltammograms confirmed the irreversibility of sufentanil oxidation [77] (Figure 7A). However, in Reference [77], it is not discussed why curves (b), (d) (Figure 7A) are negative in a wide potential range during the positively going scan.

The described EME procedure leads to the preconcentration of positively charged compounds only, so it can be used to minimize the effect of drug-protein interactions and interferences in real urine or plasma samples. The developed method offers an easy procedure for the determination of sufentanil with good selectivity, acceptable *LDR* (0.064–3.6 µmol L^−1^), and low *LOD* (20 nmol L^−1^), suitable for clinical use [77].

## 5. Conclusions

This manuscript outlined the current progress in electroanalysis of fentanyl and its analogs and metabolites, the use of suitable methods for sample pre-treatment, and provides a foundation for the development of novel sensors and methods.

The global increase of cases of fentanyl and its analogs overdosing requires the necessity to develop new, fast, inexpensive, and reliable methods for their determinations. They can be useful for appropriate drug identification and prompt medical interventions.

Based on information summarized in this manuscript, it is possible to conclude that the mentioned electrochemical methods used alone or in hyphenation (combination) with, e.g., high-performance separation methods, can be utilized for the identification and determination of fentanyl and its analogs in various types of matrices (biological and other liquid or solid matrices). The reached *LOD*s (from 2 nmol L^−1^ to 10 µmol L^−1^) and *LDR*s are comparable with those achieved by other frequently used non-electrochemical methods. These parameters are sufficient for most medical, pharmaceutical, and forensic applications. Advantages of electrochemical methods consist mostly in low production costs, simple device construction, fast responses, high sensitivity, high selectivity in the presence of common interferents, and the applicability of disposable detectors preventing cross-contamination of analyzed biological samples. Finally, hyphenated methods with microcatheter or membrane microextraction allow very simple sensors to directly analyze blood or other highly complex biological matrices, overcoming the arguably biggest limitation of the electroanalytical methods.

## Figures and Tables

**Table 1:** Structures of the commonly abused fentanyls.

Common Name (Summary Formula) Structural Formula	Common Name (Summary Formula) Structural Formula	Common Name (Summary Formula) Structural Formula
3-fluorofentanyl (C_22_H_27_FN_2_O) 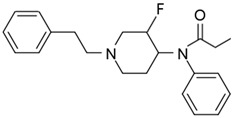	butyrfentanyl (C_23_H_30_N_2_O) 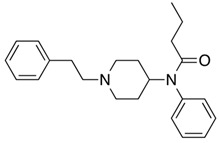	furanylfentanyl (C_24_H_26_N_2_O_2_) 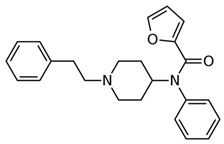
4-fluorobutyrfentanyl (C_23_H_29_FN_2_O) 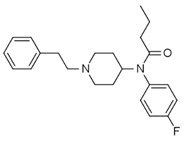	despropionyl fentanyl (4-ANPP) (C_19_H_24_N_2_) 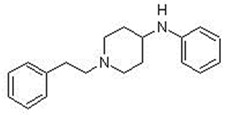	isobutyrfentanyl (C_23_H_30_N_2_O) 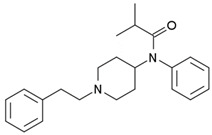
4-methoxybutyrfentanyl (C_24_H_32_N_2_O_2_) 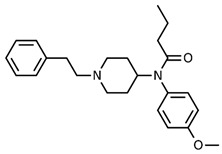	despropionyl-2-fluorofentanyl (C_19_H_23_FN_2_) 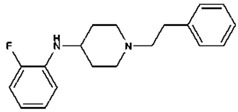	4-fluoroisobutyrfentanyl (C_23_H_29_FN_2_O) 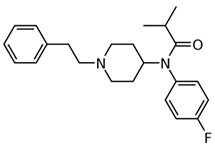
acetylfentanyl (C_21_H_26_N_2_O) 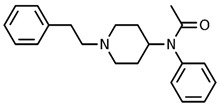	sufentanil (C_22_H_30_N_2_O_2_S) 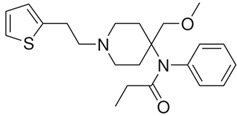	methoxyacetylfentanyl (C_22_H_28_N_2_O_2_) 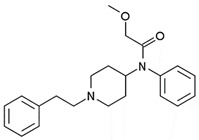
β-hydroxythiofentanyl (C_20_H_26_N_2_O_2_S) 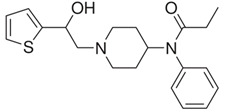	carfentanyl (C_24_H_30_N_2_O_3_) 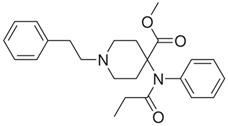	ocfentanyl (C_22_H_27_FN_2_O_2_) 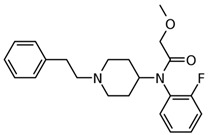
tetrahydrofuranylfentanyl (C_24_H_30_N_2_O_2_) 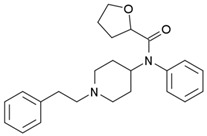	valerylfentanyl (C_24_H_32_N_2_O) 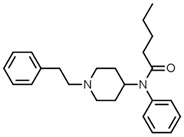	alfentanil (C_21_H_32_N_6_O_3_) 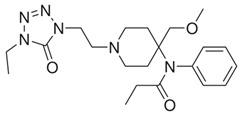
remifentanil (C_20_H_28_N_2_O_5_) 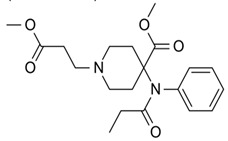	thiofentanyl (C_20_H_26_N_2_OS) 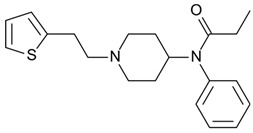	acrylfentanyl (C_22_H_26_N_2_O) 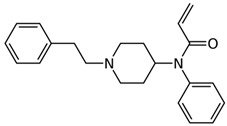
3-methylcrotonylfentanyl (C₂₄H₃₀N₂O) 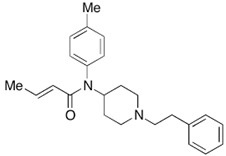	furanylbenzylfentanyl (C_23_H_24_N_2_O_2_) 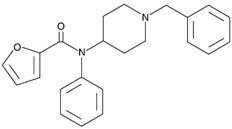	4-fluorocyclopropylbenzylfentanyl (C_22_H_25_FN_2_O) 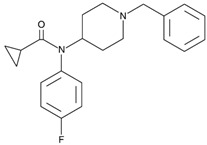

**Table 2:** Physicochemical parameters of the most important fentanyl analogs.

Compound	Mol. Mass [g mol^−1^]	Dissociation Constant p*K*_A_	PARTITION Coefficient Log *P*	Solubility in Water [g L^−1^]
Fentanyl	336.471	8.99 (DB, e)	4.05 (DB, e)	0.74 (DB, e)
		8.4 [36]	4.12 (DB, p-AG)	0.15 (p-SF)
		8.92 ± 0.20 (p-SF)	3.82 (DB, p-CA)	
		8.99 [32]	3.683 (p-SF)	
		8.44 ± 0.05 [37]	2.3 (pH 7.4 [33])	
		8.43 [38]		
Norfentanyl	232.321	9.81 ± 0.10 (p-SF)	1.59 (CS, p-ACD/LogP)	7.4 (p-SF)
			1.667 (p-SF)	
Sufentanil	386.552	8.86 (DB, p-SF)	3.95 (DB, e)	0.076 [39]
		8.51 [32]	3.4 (DB, p-AG)	0.012 (DB, p)
		8.01 [39]	3.61 (DB, p-CA)	0.15 (p-SF)
		8.0 [36]	3.950 (p-SF)	
		7.89 ± 0.20 (p-SF)		
Carfentanyl	394.515	8.05 (DB, p-CA)	3.7 (DB, p-AG)	0.0259 (DB, p-AG)
		7.76 ± 0.20 (p-SF)	3.67 (DB, p-CA)	0.19 (p-SF)
			3.684 (p-SF)	
Acetylfentanyl	322.44	8.92 ± 0.10 (p-SF)	3.173 (p-SF)	0.30 (p-SF)
alfa-methylfentanyl	350.50	9.37 ± 0.20 (p-SF)	4.49 (DB, p-AG)	0.014 (DB, p-AG)
			4.23 (DB, p-CA)	
Acrylfentanyl	334.45	8.72 ± 0.10 (p-SF)	3.201 (p-SF)	0.037 (p-SF)
Butyrfentanyl	350.50	8.92 ± 0.20 (p-SF)	4.44 (DB, p-AG)	0.0137 (DB, p-AG)
			4.26 (DB, p-CA)	
Cyclopropylfentanyl	348.48	8.75 ± 0.10 (p-SF)	3.564 (p-SF)	0.045 (p-SF)
Furanylfentanyl	374.48	8.71 ± 0.10 (p-SF)	5.277 (p-SF)	0.012 (p-SF)
Methoxyacetylfentanyl	352.47	8.88 ± 0.20 (p-SF)	2.574 (p-SF)	0.85 (p-SF)
Ocfentanyl	370.46	8.81 ± 0.20 (p-SF)	2.816 (p-SF)	0.26 (p-SF)
tetrahydrofuranylfentanyl	378.51	8.71 ± 0.10 (p-SF)	2.815 (p-SF)	0.016 (p-SF)
*p*-fluroisobutyrylfentanyl	368.49	8.91 ± 0.20 (p-SF)	4.150 (p-SF)	0.027 (p-SF)
Alfentanil	416.52	7.82 ± 0.20 (p-SF)	2.16 (DB, e)	0.252 (DB, p-AG)
			2.2 (DB, p-AG)	
			2.81 (DB, p-CA)	
Remifentanil	376.45	6.65 ± 0.20 (p-SF)	1.75 (DB, p-AG)	0.591 (DB, p-AG)
			1.52 (DB, p-CA)	

DB = DrugBank (DrugBank, Edmonton, Alberta, USA), CS = ChemSpider (Royal Society of Chemistry, UK), PC = PubChem (National Center for Biotechnology Information, USA), SF = SciFinder (p*K*a—the most basic; 25 °C) (American Chemical Society, Washington, D.C., USA) e—experimental value, p—predicted value, CA—ChemAxon (ChemAxon, Budapest, Hungary), AG—ALOGPS (Helmholtz Zentrum München, Munich, Germany), ACD/Log*P*—Advanced Chemistry Development software/Log*P* (Advanced Chemistry Development, Toronto, Canada). p-SF: Calculated using Advanced Chemistry Development (ACD/Labs) Software V11.02 (© 1994–2020 ACD/Labs) (Advanced Chemistry Development, Toronto, ON, Canada).

**Table 3:** Overview of electroanalytical methods used for fentanyl and its derivatives determination.

Analyte	Method	Working Electrode/Detector	*LOD* [µmol L^−1^]	Ref.
DBPPE	ASV	SMDE	0.005	[65]
Fentanyl	ASV	SMDE	0.050	[66]
Fentanyl	DPV	SPCE modified with MOF	0.3	[67]
Fentanyl	DPV	MWCNTs-GCE	0.1	[68]
Fentanyl	DPV	GCE + carbon nano-onions	0.3	[49]
Fentanyl	DPV	SWCNTs	0.0011	[69]
Fentanyl, norfentanyl	SWV	MWCNTs	0.05	[70]
Fentanyl	SWV	Microcatheter-based dual sensor	0.00218	[71]
Fentanyl	SWV	SPCE	10	[72]
Fentanyl	SWASV	SPCE	0.110 ± 0.051	[73]
Fentanyl	SWASV	SPCE	0.692 ± 0.074	[73]
Fentanyl	Potentiometry	ISME	5.43	[63]
Fentanyl	Potentiometry	ISME	6.29	[74]
Fentanyl	HPLC	AD	1.3	[9]
Fentanyl analogs	HPLC	AD	1.3–8.7	[9]
Fentanyl	ECL	GCE and ILCPE	0.0085	[75,76]
Sufentanil	DPV	SPE	0.020	[77]

Analytes: DBPPE—N,N′-bis(1-phenylmethyl-4-piperidinyl)-ethane-diamide. Methods: ASV—anodic stripping voltammetry, DPV—differential pulse voltammetry, SWV—square wave voltammetry, SWASV—square wave anodic stripping voltammetry, HPLC—high-performance liquid chromatography, ECL—electrochemiluminescence. Detectors: AD—amperometric detector, SMDE—static mercury drop electrode, CPE—carbon paste electrode, GCE—glassy carbon electrode, SPE—screen-printed electrode, SPCE—screen-printed carbon electrode, ISME—ion-selective membrane electrode, SWCNTs—single-walled carbon nanotubes, MWCNTs—multi-walled carbon nanotubes, MOF—metal-organic framework, ILCPE—ionic liquid composite paste electrode.

## Data Availability

Not applicable.
